# Suppression of crosstalk in multielectrode arrays with local shielding

**DOI:** 10.3389/fnano.2022.948337

**Published:** 2022-08-04

**Authors:** J. R. Naughton, J. A. Varela, T. J. Connolly, S. Shepard, T. E. Dodge, K. Kempa, M. J. Burns, J. P. Christianson, M. J. Naughton

**Affiliations:** 1 Department of Physics, Boston College, Chestnut Hill, MA, United States,; 2 Department of Psychology and Neuroscience, Boston College, Chestnut Hill, MA, United States,; 3 Department of Biology, Boston College, Chestnut Hill, MA, United States,; 4 Integrated Sciences Nanofabrication Clean Room Facility, Boston College, Chestnut Hill, MA, United States

**Keywords:** multielectrode array, extracellular, optogenetics, nanofabrication, crosstalk

## Abstract

Electrical crosstalk can constrain the performance of multielectrode arrays in electro- and neurophysiology, in terms of both stimulation and recording. This is especially so at high electrode density, desirable for spatiotemporal mapping of bioelectrical signals from multiple cells. Channel interference due to crosstalk is currently only partially addressed, *via* continuous interleaved sampling or post-data acquisition spike sorting. Here, we show that a locally-shielded electrode architecture significantly suppresses crosstalk, and enables multi-site recording at high electrode density without the need for spike sorting. Arrays of shielded electrodes, prepared by micro- and nanofabrication techniques in a vertically-oriented coaxial geometry, demonstrate at least a 400 times improvement in spatial density over the unshielded case.

## Introduction

The brain contains both large- and small-scale spatiotemporal organization, with different functions taking place on multiple spatial and temporal scales. To gain insight into the rules that underlie brain function, it is generally accepted that networks of neurons need to be studied (Nadasdy et al., 1998), as opposed to individual neurons in isolation. The extracellular multielectrode array (MEA) is an appropriate device for stimulation of and recording from large numbers of neurons (as well as other electrogenic cells), as it is capable of simultaneously recording both the slow activity associated with changes in the local field potential (an aggregate of the surrounding synaptic inputs) and the fast activity associated with multi-unit neuronal discharges nearby. In order to gain a mechanistic description of the biophysical contributors to neuronal network processes, it is necessary to isolate single-unit activity. Given the size and spacing of neurons within networks [on the 1–10 μm scale (Heathcote and Sargent, 1987)], this requires a device capable of high spatial resolution. While MEAs can be fabricated at high densities [down to 10’s of μm pitch (Multichannel Systems, 2021; Miccoli et al., 2019)], a limiting factor in achieving higher spatial resolution is isolating the activity of individual neurons within the larger array (Ventura and Gerkin, 2012).

Due the spread of the extracellular current into surrounding ionic medium originating from action potentials, the number of effective recording channels in a device will be reduced below their actual number if they are of a critical spacing or smaller. The extent to which an electric field originating from a neuron is recorded by multiple electrodes, rather than by/at a single recording site, may be defined as electrical crosstalk (Wilke et al., 2011). The reciprocal of this situation, coinciding fields from multiple neurons at a single recording site (which then aggregate as a single input instead of multiple distinct inputs) is equally problematic. Overlapping electric potentials and fields are undesirable for both recording and stimulation, the latter being identified as an issue in, for example, MEA technology used for visual prostheses (Nelson et al., 2017). One definition of the degree to which a single pixel of an electrode array dominates all neighboring pixels was given by Wilke, et al. (2011) as the crosstalk coefficient *CT* = *E*_*N-1(x,y,z)*_/*E*_N(x,y,z)_, where |*E*|*N(x,y,z)* is the electric field magnitude at a point (*x, y, z*) with all electrodes in the array active, and |*E*|_*N-1(x,y,z)*_ is the field at that point with a chosen electrode inactive. This ratio will be the same for electric potential (voltage). *CT* ranges from 0 to 1, with the low end being minimal crosstalk (measured electric field dominated by the measuring electrode of interest) and the upper end being high crosstalk (multiple electrodes contributing to the measured electric field).

If the overlap between neuronal events (spikes) is relatively small, a common tool used by neuroscientists to isolate individual neurons in multi-unit recordings is the post-data acquisition process of spike sorting. This involves grouping recorded spikes into clusters based on the similarity of their waveforms (Rey et al., 2015) (i.e., voltage dynamics over time). Spike sorting extracts individual spike waveforms out of a temporal window of collected spikes and is highly dependent on the sampling rate. Each datum point of a spike is a possible “feature” to be used for differentiation from other spikes and therefore the problem starts off being an N-dimensional one, where N is the number of data points per spike. Here, the complexity increases exponentially as a function of recorded events and can quickly becomes computationally intensive (Prentice et al., 2011; Hilgen et al., 2017).

The duration of an action potential is on the order of a few milliseconds and so a typical 40 kHz sampling rate will yield 50–100 points per spike. If the sampling rate is too low, it risks becoming insufficient, as cutting out data points can cause an unintentional shift in the maximum point used for alignment. A higher sampling rate corresponds to more data points and a higher accuracy in representing the signal, but requires more computational power. To lower this burden, methods have been developed in order to lower the dimensionality of the problem. One simple method for feature extraction is to take the basic characteristics of a waveform (amplitude, duration/width, rise time, square of the signal, etc.) and use them to differentiate signals. However, it has been shown that this is not always reliable (Lewicki, 1998). Another simple approach called template matching relies on choosing template spike shapes for each unit (Gerstein and Clark, 1964). The shape is then used as a metric in assigning and matching waveforms. However, in addition to manual intervention being problematic, sparsely-firing neurons could be missed with this approach (Quiroga et al., 2007), and action potentials in many types of neurons exhibit intrinsic plasticity evident as experience-dependent changes in membrane ionic conductances and corresponding changes to the dynamics of the action potential (Debanne et al., 2019). The most common feature-extraction and dimensionality-reduction method is principal component analysis (PCA) (Harris et al., 2000; Shoham et al., 2003). While the details of this method are beyond the scope of this paper, the idea is to find an ordered set of orthogonal basis vectors that captures the directions of largest variance in the data and represents any waveform as a linear combination of those principal components (Person, 1901; Quiroga, 2013). Despite these challenges, spike sorting algorithms currently remain a standard process in analysis of neurological data and new methods or refinements are continually being made (Ekanadham et al., 2014; Kadir et al., 2014; Swindale and Spacek, 2014). However, establishing metrics for evaluating spike sorters is an on-going process, with some research showing there is no one-size-fits-all algorithm (Buccino et al., 2020; Magland et al., 2020; Hall et al., 2021).

The most challenging issue to the spike sorting method is the subject of focus for this paper: overlapping spikes or, as defined above, crosstalk. Two or more neurons in close proximity firing synchronously or with a small enough delay will have overlapping extracellular action potentials. This could be interpreted as a signal from a single neuron, rather than from distinguishable neurons. Furthermore, the extracellular waveform originating from an action potential changes shape as it travels through space. Given that field potentials can travel hundreds of microns in ionic solution, the waveform picked up at one location could be drastically different at another and therefore incorrectly interpreted as two unique signals (Einevoll et al., 2012). Outside of spike sorting, various techniques have been utilized to try to minimize the effect of crosstalk by designing devices that constrain the generated electric fields (Chai et al., 2008; Wong et al., 2009; Moghaddam et al., 2011; Kaur et al., 2021).

**FIGURE 1:**
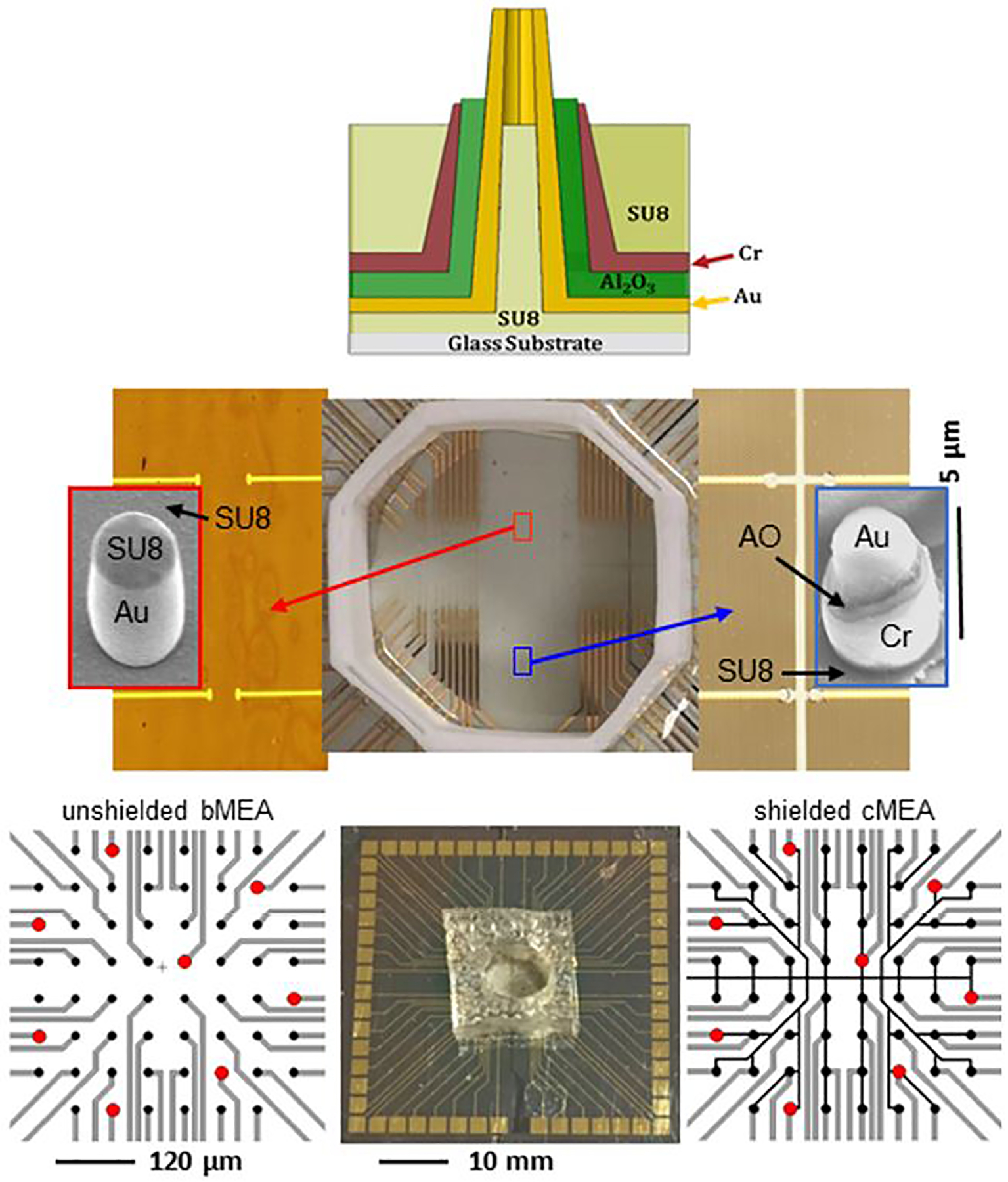
Multielectrode array devices used for electrical and optical stimulation. Upper: Schematic of coaxial electrode comprising the cMEA device. Middle: Electrical stimulation device (center) with unshielded, bare electrode/bMEA (left) and shielded, coaxial electrode/cMEA (right) regions. Left and right are magnified views of the respective regions, each containing rows of sensing areas with varying separation. The sensing areas are the 10 μm in diameter circles at the ends of gold address lines. All areas outside of sensing areas have been passivated (insulated). Zoomed insets show SEM images of respective individual, ~2 μm diameter, vertically-oriented electrodes. Constituent material labeled (AO = Al_2_O_3_). Lower: Optical stimulation device with cMEA chip with well (center), and wiring schematics for bMEA (left) and cMEA (right), both with 10 mm diameter sensing regions (black dots) at 60 mm pitch. Red dots indicate 20 mm-diameter transparent areas on the substrate and under the sensors, facilitating optical illumination up through the electrode cores.

In this paper, we show that local electrical shielding through a coaxial structure (Naughton et al., 2016) greatly reduces crosstalk when compared to the conventional bare, unshielded electrode. Two stimulation methods, electrical and optical, are employed to demonstrate the utility of local shielding versus the unshielded case, *via* electrode recordings of stimulated voltage transients. In the optical stimulation experiment, optogenetically-transfected human embryonic kidney cells (HEK 293-ChR2) were employed, as proxies for electrogenic cells such as neurons. Two types of devices were fabricated for each stimulation method: bare multielectrode arrays (bMEA) and coaxial multielectrode arrays (cMEA). Figure 1 upper shows a schematic of an individual coaxial electrode, with its constituent materials. In Figure 1 middle, we show optical images of the wiring layout for the electrical device (bare = red arrow, coax = blue arrow), and electron microscope images of an individual electrode for each type. In Figure 1 lower, we show the wiring layouts for the optical devices, and a photograph of an actual chip. We indicate representative locations of clear holes (red dots) in an otherwise optically-opaque metal film on the glass substrates onto which the MEAs (pixels = black dots) were fabricated. These holes enabled localized optical illumination and excitation *via* from below. In each cMEA stimulation device, the outer conductors/shields of the coaxes were common, with the cores individually electrically-addressed.

## Results

### Simulations

We also modeled/simulated the performance of unshielded and shielded MEA configurations. Using the finite element method (FEM) simulation software COMSOL Multiphysics (RRID: SCR_014767), a computational model of the device was made employing realistic material parameters, intending to examine the overlap of electric potential of a pair of electrode sensing areas as a function of electrode separation. A pattern of seven rows of electrode pairs, arranged with each row having a specific separation distance (from 5 μm to 1,000 μm), was placed in a simulated electrolyte solution (having nominally the same electrical properties as the medium used in the electrical experiment, i.e., static dielectric constant *ε* ~ 80, dc electrical conductivity *σ* ~ 1.5 S/m). Although crosstalk and the detection of field potentials *in situ* are influenced by a myriad of factors including cell type, distance from electrode and the nature of the contact with electrodes, the purpose of this simulation was to find the amplitude of the potential at the recording electrode surface generated by a source (e.g., neuron spike) as a function of separation distance. Green-Lorentz reciprocity reduces this problem to solving Poisson’s equation for the scalar potential generated from the recording electrode as a voltage source (Lorentz, 1896).

**FIGURE 2:**
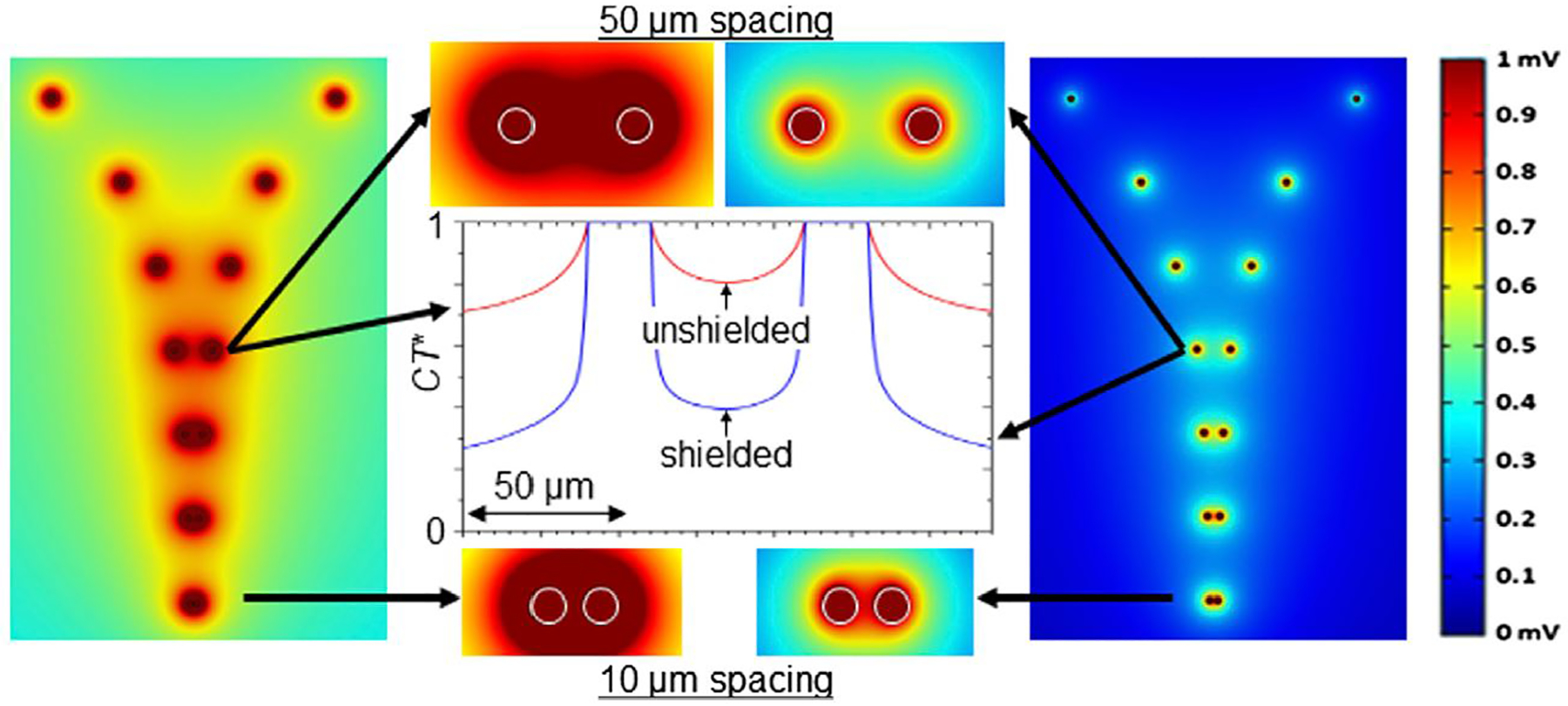
Finite element modeling. Top view of simulated equipotential contours for (left) bare and (right) shielded electrodes (5 μm height, 20 μm-diameter), biased at 1 mV, with electrode spacings, from the top of 1,000, 500, 250, 100, 50, 25, and 10 μm. Color bar at far right indicates strength of signal from a source (e.g., action potential/neuron spike) sensed by an electrode. Middle panel shows magnified view of 50 and 10 μm spacing rows, as well as calculated CT* values versus distance for the 50 μm spacing. The coaxial, shielded electrodes include simulated radially coatings of 200 nm Al_2_O_3_ and 120 nm Cr, the latter with height 3 μm.

Simulations were performed for bare, unshielded and coaxial, shielded electrodes for a range of electrode diameters and heights, as well as, for the shielded case, with an outer shield of various heights relative to that of the core electrode. Shown in Figure 2 are results for 20 μm-diameter, 5 μm-tall electrode pairs, with the shields in the coaxial case 60% the height of the core (i.e., 3 μm). Experiments were later performed with bare electrodes and coaxial electrodes having such 60% shielding. For clarification, the simulations of pairs were performed separately for each separation distance, all with 1 mV excitation. The 10 and 50 μm separation results are expanded in the middle panel of Figure 2. Throughout, dark red (blue) represents regions where an electrode strongly (weakly) senses the source signal, as indicated by the color scale at right. It is clear from the images that unshielded electrodes (left images) experience overlap in the sensing regions of adjacent electrodes at separation distances far greater than do the shielded electrodes (right images). For distances of 50–100 μm and less, the sensing regions of the unshielded electrodes appreciably overlap. This effectively renders two individual electrodes as a single electrode of larger size, representing a loss in pixelation density and thus an emergence of crosstalk. Conversely, even at separation distance as small as 10 μm (bottom of middle panel in Figure 2), the shielded electrodes continue to show a separation of signal with CT* ≤ 0.8. Here, CT* = |*V*(*d*)|/|*V*(0)|. CT*is an effective crosstalk coefficient and |*V*(*d*)| is the signal of a particular sensing region a distance *d* from the excitation source. Simulation results for CT*, from linear cuts through neighboring electrodes at 50 μm spacing, are shown in the center panel of the figure.

It is important to note that these simulations are largely scale invariant. That is, if the bare electrodes were of smaller or larger diameter than those simulated in Figure 2, the sensing regions would overlap and still be dominated by crosstalk at separation distances of a few times the electrode diameter. Multiple 2D and 3D geometries have been simulated and the results are qualitatively the same. Since the goal of this work is to move to higher density arrays eventuating in a sensing region comprised of a single coax, we made the geometry of the simulation as to compare a single coaxial structure with conventional MEA technology (a single, flat, cylindrical pad). While our experimental arrays feature multiple coaxial structures within a single sensor region, the simulations represent a conservative estimate of field overlap since the experimental arrays have more shielding surface area. For comparison, in conventional MEA architectures, the field distribution will likely be larger because of the lack of local shielding and greater surface area found in a capped cylinder (as opposed to the coaxial structure which has the cap removed). Therefore, the results of this simulation can give one a sense of the maximum pixelation allowed, given an electrode size, in order to avoid a large amount of crosstalk.

### Electrical and optical stimulation

**FIGURE 3:**
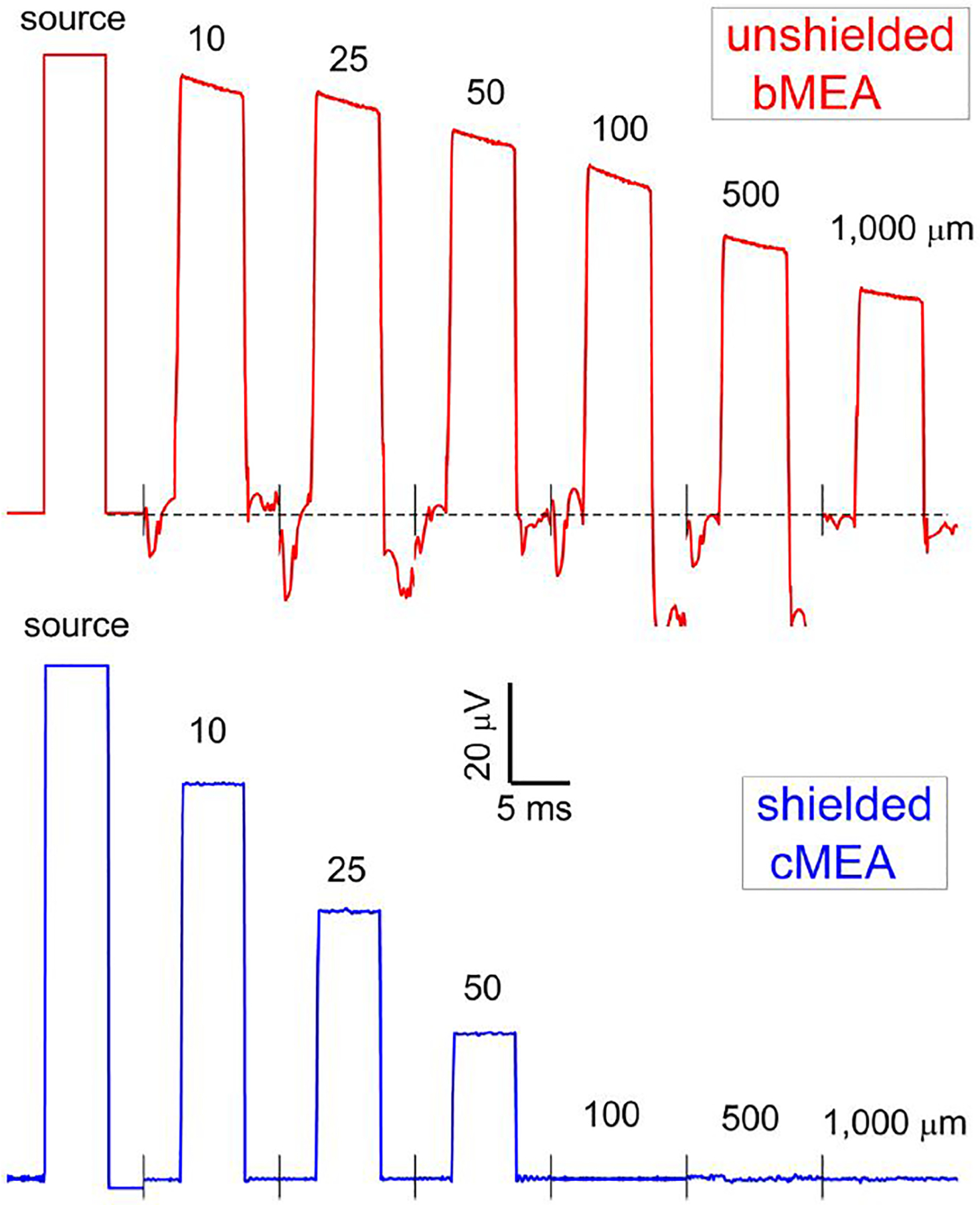
Recording with bMEA and cMEA under electrical excitation. Voltage pulse of (100 μV, 5 ms) as applied to one electrode in the arrays of Figure 1-upper indicated as source, with recorded waveforms of the bMEA (upper) and cMEA (lower) at distances from source as shown.

For electrical stimulation experiments, the well of the device in Figure 1 (middle), containing independent bMEA and cMEA regions, was filled with an electrolyte buffer solution using a pipette. Starting with the bMEA device and the 1 mm separation row, and moving incrementally to the 5 μm separation row, a voltage pulse was sent to one left electrode (using a Ag/Cl pellet in the electrolyte buffer solution as a ground), with voltage signals at all remaining electrodes recorded simultaneously. The experiment was repeated using the right electrode of a particular row as the stimulating electrode, to ensure mirror symmetry. Raw data traces for distances from excitation source *d* = 10, 25, 50, 100, 500, and 1,000 μm are shown in Figure 3 (upper, in red). For tests on the cMEA, one coax location’s shield (rather than a distant wire) was set to ground. The same procedure as above was performed (signal injected on the left, followed by the right, to confirm symmetry). Raw cMEA data traces are shown in Figure 3 (lower, in black), again for *d* = 10–1,000 μm. Data from five-sweep trials were averaged and the mean values plotted with error bars showing the dispersion in the steady-state value. It was observed from experiments that signals recorded during the first sweep were highest, while subsequent sweeps moved towards a steady state value.

**FIGURE 4:**
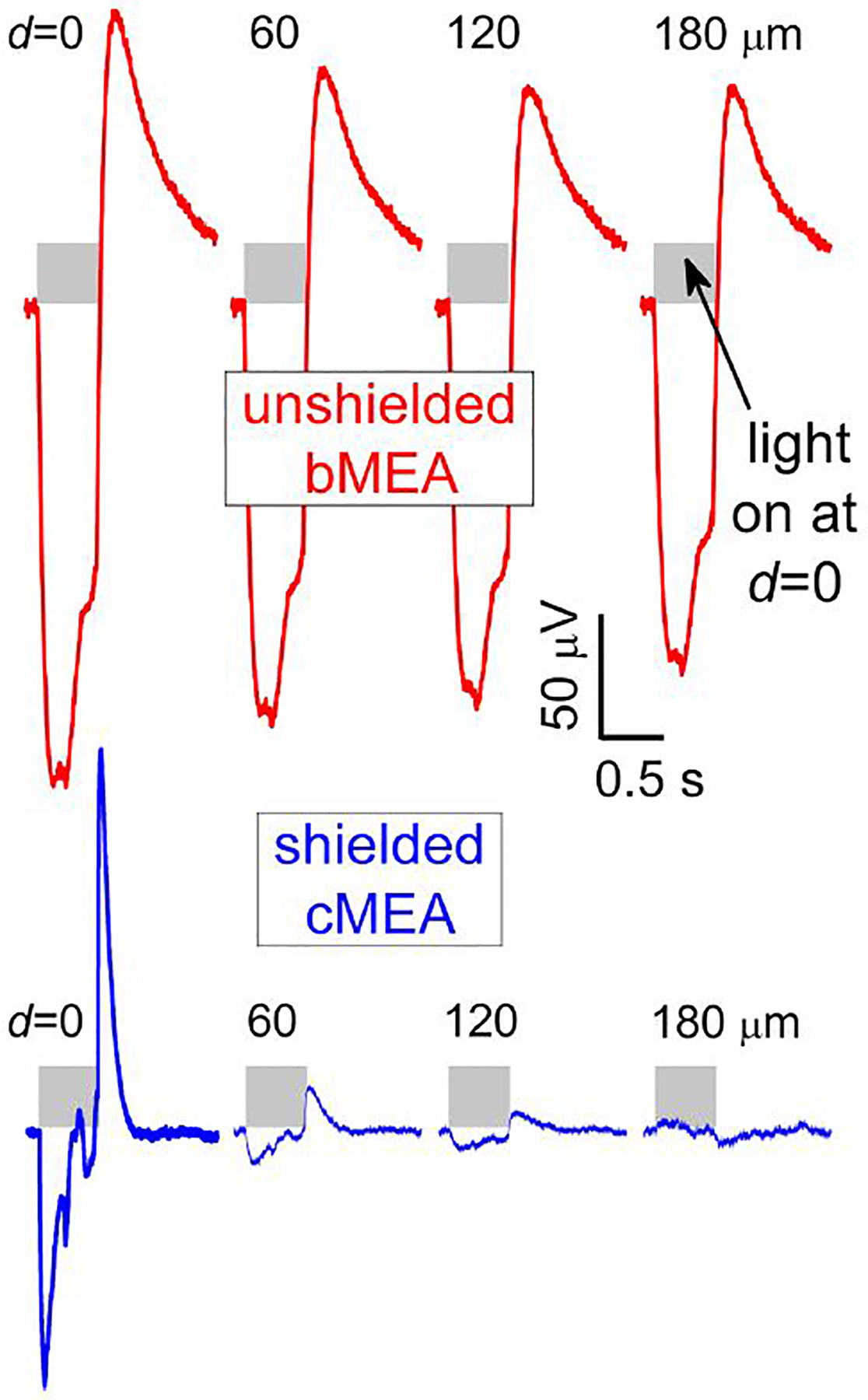
Recording with bMEA and cMEA under optical excitation. Voltage response vs. time of optogenetically-transfected HEK-293 cells for the bMEA (upper) and cMEA (lower) devices in sensing regions at indicated distances *d* from backside-illuminated (0.5 s duration, shaded regions) stimulation location (*d* = 0). Large extracellular potentials were evident at the site of stimulation and nearby electrodes in the bMEA but only at the site of stimulation on the cMEA. All windows correspond to 1.5 s recordings.

For optical stimulation, optically-evoked field potentials were detected in HEK-293 cells transfected with the blue-light sensitive channelrhodopsin protein ChR2(H134R) (Zhang et al., 2007). Transfection and culture processes on the arrays, described in previous work (Naughton et al., 2016), were used. Devices were aligned in the measuring apparatus amplifier (Multichannel Systems MEA-2100) and one of the 60 channels was set to ground. To ensure one had live, working cells throughout the array (i.e., capable of optical actuation), a 473 nm wavelength laser was aligned above an array and illuminated the sample. Once cell viability was confirmed *via* captured deflections, the laser was moved to backside alignment, sequentially positioned to several sites below the array, and five-sweep trials were performed. Representative raw data plots of voltages at the source (i.e., a cell excited at the illuminated coax) and at multiple channel distances from that source are shown in Figure 4, for both the bMEA (upper) and cMEA (lower) devices. It can be seen there that, for each light-excitation site, the deflections in the local field were confined to only a few proximate (i.e., illuminated) sensing regions for the cMEA, as opposed to nearly full signal at least 180 μm away in the bMEA. We note that the waveforms of the optically-induced deflections in HEK cells seen by the cMEA appear somewhat different from those seen by the bMEA. Both the bare electrodes and the cores of the shielded array are capacitively coupled to the signal from the cells, but the shielding in the latter serve to significantly reduce this coupling. Assuming the same liquid medium impedance *R* for both structures, this serves to correspondingly reduce the time constant *τ* = *RC* of the cMEA measuring circuit. Specifically, the data in Figure 4 yield *τ* ~ 475 and 120 ms for the bMEA and cMEA, respectively. We have simulated the capacitance *C* of these circuits using electromagnetic finite element modeling routines (CST Studio Suite), and calculate a capacitance ratio of *C*_bMEA_/*C*_cMEA_ = 5 ± 2, consistent with the measured ratio of ~4. As such, the cMEA structure better represents the extracellular true response.

For both electrical and optical stimulation, the recorded electrical response was confined to sensing areas in close proximity to the stimulation location for the cMEA device. On the other hand, this response persisted to significantly larger distances for the bMEA device. In Figure 3, electrical stimulation, it can be seen that the response is ~75% suppressed a distance of 50 μm from the source for the cMEA, but only ~15% suppressed for the bMEA case. Beyond that distance, cMEA electrodes detect no residual signal, while almost half the stimulation signals persist out to 1 mm distance in the bMEA. Both electrical and optical stimulation results reflect the suppression of channel crosstalk in the shielded environment provided the cMEA.

## Discussion

**FIGURE 5:**
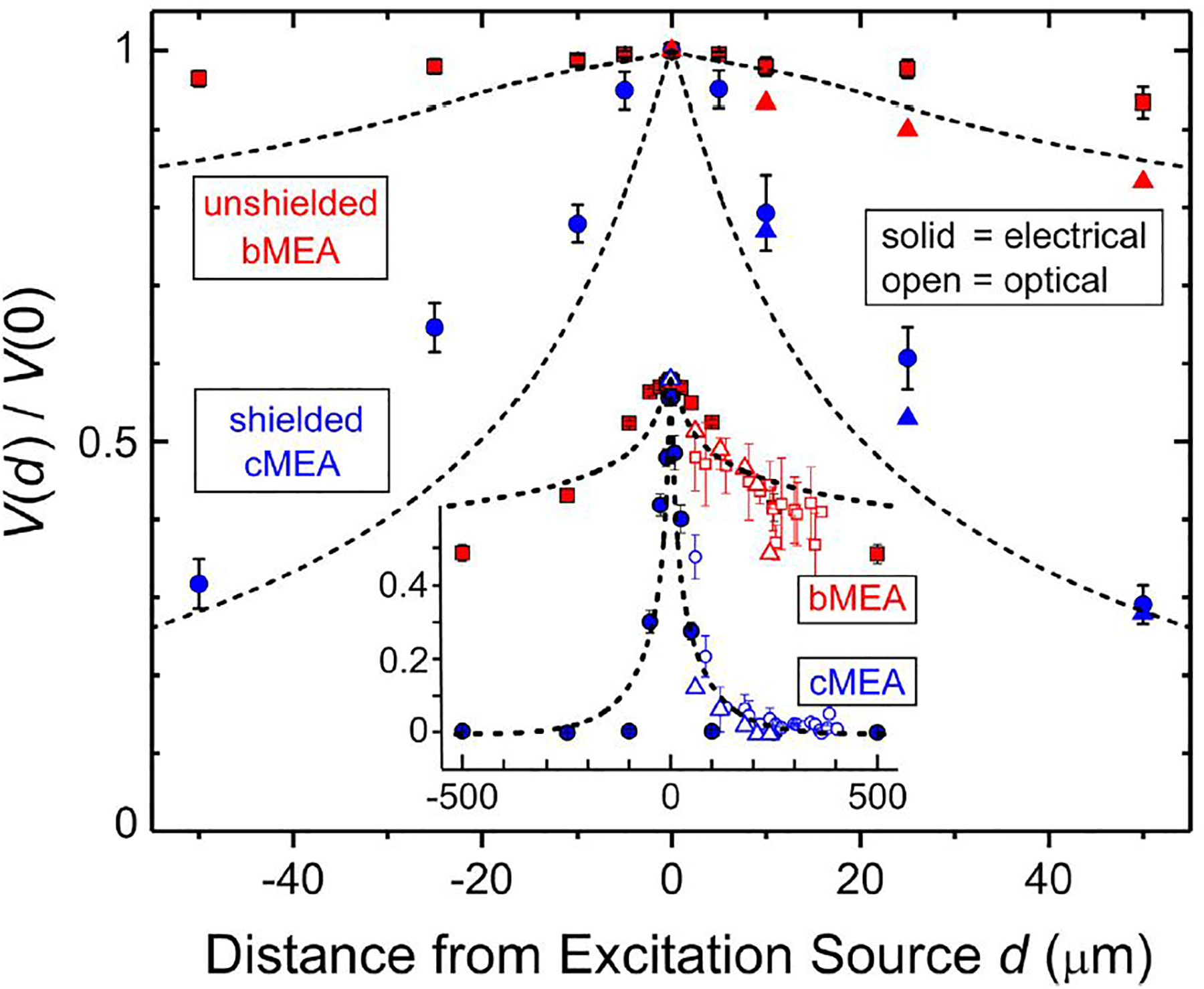
Comparison of excitation and device types. Main panel: Effective crosstalk coefficient *V*(*d*)/*V*_o_ vs. distance *d* from excitation source for electrical excitation experiments, for both unshielded bMEA (red symbols) and shielded cMEA (blue symbols) devices. Inset: Wider range of data, out to *d* = 500 μm, for both electrical (solid symbols) and optical (open symbols) stimulation. Standard deviations are indicated. Lines are results from simulations of electrical response versus *d*, for both types of devices. Different symbols for same conditions refer to results on different identical devices. Data from five-sweep trials were averaged and mean values plotted with error bars showing ± standard deviation.

In order to quantitatively compare the two devices, data were collected and trials for each experiment averaged. The electrode voltage at the point of excitation was named *V*(0). The distances to the surrounded electrodes were calculated and the crosstalk coefficient CT*, introduced above, was extracted for each electrode. Like the *CT* discussed in the introduction, a large CT* corresponds to high crosstalk, since the sensing region is capturing a large portion of the source. We show in Figure 5 the voltage response, plotted as effective crosstalk CT* = *V*(*d*)/*V*(0), versus distance *d* from excitation for the electrical and optical stimulation experiments, from data in Figures 3, 4 as well from as additional like experiments (averaged data ± standard deviation shown). Red (blue) symbols are data from unshielded bMEA (shielded cMEA) samples, open (solid) symbols are from optical (electrical) experiments. The dashed lines represent the computer simulated response using the same formalism as employed for Figure 2. Data sets from two of each type of MEA device are shown (thus the two symbol types for each). By combining the optical and electrical stimulation data sets, one sees a common trend, in that for both experiments, the coax outperforms bare electrodes in reducing crosstalk. These results are summarized in Figure 5, where one can see that the effective crosstalk coefficient for the shielded electrodes is significantly lower than that for the bare electrodes for all distances greater than a few micrometers from the source. This corresponds to significantly better suppression or filtering of stray electric fields from sources far from the recording device. In the bare electrode devices, sensing regions within 100 μm of the signal still show a CT* more than 0.9, which corresponds to very large crosstalk. Such signal overlap might become problematic from the perspective of establishing ground-truth data. One can get a quantitative sense of the effect of local shielding in MEA technology from Figure 5. That is, using 50% crosstalk as a representative gauge, the shielded, coaxial case yields a ~20× improvement in linear spatial resolution (~25 vs. 500 μm for unshielded, Figure 5). This corresponds to a 20^2^ ~ 400× improvement for an areal device, e.g., versus a typical MEA.

The main limitation of this study is the selected cell culture used in the optical stimulation experiment. The technical ease in achieving transfection for optogenetic applications, high density to ensure electrode coverage, electrical characteristics, and high survivability of HEK cells make them a suitable choice in a proof-of-concept study. However, there is an obvious difference between HEK cells and neurons such as those typically cited in neural studies. An appropriate next step would be to culture transfected neurons or hippocampal slices. This would further develop the proof-of-concept on two dimensions: 1) showing crosstalk reduction in cells with well-known electrical characteristics and thus able to verify ground-truth measurements, and 2) testing the morphology dynamics of the cell-coaxial electrode interface. Moving from a quasi-2D system, as that found in conventional MEA technology, to a 3D architecture could help prevent cell drift, while at the same time, promote cell engulfment of the inner electrode, which has shown the ability to record subthreshold signals and, coupled with electroporation, intracellular activity by penetrating the cell membrane. We do not, however, anticipate that different cell lines will have a profound effect on the results.

## Summary and conclusion

The experiments and simulations presented herein demonstrate the suppression of crosstalk enabled by local shielding through the use of a coaxial electrode architecture. They suggest that, in order to avoid signal overlap in high density multielectrode arrays, a coaxial or similarly locally-shielded architecture could be utilized. While current state-of-the-art electrode arrays (e.g., HD MEAs, CMOS-based multiplexing sensor arrays, neuropixels) achieve high density, crosstalk represents a rate-limiting effect on spatial resolution. Conversely, the present coaxial architecture, in addition to achieving even higher pixel density than presented here (nothing in the fabrication process prevents us from pushing the pixel density into the nanoscale), is able to reduce electrical crosstalk and thus maintain high spatial resolution. Theoretically, the shielded architecture can improve until the thermal noise floor becomes dominant. For nanoscale coaxial arrays, using the Johnson-Nyquist noise equation as a first order approximation, one should still have a practical S/N ratio with a noise floor of tens of microvolts. The device characteristics could be further improved by the changing the impedance through increasing the inner electrode surface area (lowering the shield) or changing the inter-electrode (inner—outer metal annulus) gap.

In the reciprocal paradigm, where electrodes are utilized for evoking cell behavior through electrical stimulation, we have shown the coaxial architecture is advantageous in achieving localized cell engagement. This advantage also extends to isolated optical stimulation. Instead of achieving targeted optical stimulation through beam steering or scanning-based approaches, the shielded electrode facilitates *in situ* optical integration. Future work in device development could also move to an entirely on-device optical component, thus allowing high speed, individually addressable optoelectronic recording and modulation. In addition to alleviating the burden of advanced optical methods, such adoption could reduce dependence on spike sorting, elaborate as algorithms for such have been developed, in analysis of multicell recordings using multielectrode/microelectrode arrays. The locally-shielded array configuration can thus find future utility in connectomic studies of neuronal and other electrogenic cell configurations.

## Materials and methods

For devices for optical stimulation experiments, a 10/300 nm Ti/Au layer, thick enough to be optically opaque in the 400–700 nm wavelength range, was sputter deposited onto glass substrates, followed by standard photolithography and wet etching to yield eight 20 μm-diameter openings (in the metal) spaced 300 μm apart. These openings were necessary to facilitate light (from a 472 nm laser), later used to evoke ion currents in HEK cells, to transmit through the glass and up through the cores of the pillars, then onto a specified region, rather than macro-illuminating the sample (to the full diameter of the light cone and thus covering multiple sensing regions). The light cone of the laser was measured to be ~300 μm in diameter in the geometry employed, such that only one region would be illuminated at a time. Nanoimprint lithography (NIL) was then used on 5 μm-thick SU8 photoresist to create a 10 mm^2^ area pillar array (containing 2 μm diameter, 5 μm tall pillars at 10 μm hcp pitch) (Rizal et al., 2013) atop this metal film. After depositing a 10/120 nm Ti/Au layer (to serve as the bMEA electrode metal or the cMEA core electrode metal), an 8 × 8 square array of 10 μm diameter sensing areas (each encompassing on average three pillar electrodes to be wired in parallel) at 60 μm pitch was patterned using photolithography, and a subsequent wet chemical etch left 60 individually addressed sensing areas (i.e., excluding the four corners). These sensing areas were aligned with the aforementioned openings in the light-confining metal layer. Atomic layer deposition (ALD) was used to deposit a 225 nm thick aluminum oxide layer covering the entire sample (thus passivating the address lines), and photolithography plus wet etching was used to open up holes over the macroscale pad (pin-out) regions.

For the coaxial structures, an outer metal layer of 120 nm thickness Cr was further deposited (to serve as the coax shield electrode) and photolithography + wet etching was used to pattern the Cr. To expose the inner coax metal as well as to decapitate the pillars to facilitate the transmission of light through the 2 μm core of each pillar in the sensing area, and thus allow for optical stimulation, two processes were used. An SU8 layer was spun on and baked to form a mechanical stabilization layer. Next, a chemical mechanical polisher was used to decapitate the pillars and standard wet chemical etching was used to lower the heights of the Cr and alumina layers around the coax pillar core. A plasma etch process was then used to lower the height (thickness) of the SU8 filler. In order for the HEK cells to be grown and contained within the electrode region, a PDMS liquid-confining well (5 mm diameter, 10 mm height) was attached to the substrate, also using PDMS.

For the electrical stimulation experiments, devices were also fabricated on glass substrates. A NIL process similar to that described above was used to create two SU8 pillar array regions (10 mm^2^ areas containing similar 2 μm diameter, 5 μm tall pillars at 10 μm pitch), with the regions separated by 50 mm. Each region comprised a 20 μm diameter sensing area, which contained on average seven individual pillars. The sensing areas were arranged as seven rows of pairs, each row having a different separation distance, from 1,000 to 10 μm. One region contained a bMEA, and the other a cMEA. A metal layer (10/110 nm Ti/Au) was deposited *via* physical vapor deposition and photolithography + wet etching was used to define the 28 individually addressed sensing areas in each of the two pillar regions (56 total areas). Next, a 200 nm thick aluminum oxide layer was deposited on the entire sample using ALD. Holes were etched in the alumina in order to access the Au layer macro pads (where the address lines originating from the sensing areas terminated) corresponding the pin locations on the pre-amplifier board. Finally, a 120 nm Cr layer was deposited using physical vapor deposition. Photolithography was used to pattern the cMEA region, so as to leave Cr covering 28 sensing areas and to have subsequent address lines coming from each area. To expose the cMEA inner metal, an anisotropic lithographic process was combined with subsequent wet etching in order to lower the heights of the Cr and alumina layers. The resulting outer metal to inner metal height ratio was ~0.6. Two plastic wells fabricated using a 3D printer were attached with PDMS to contain an electrolyte buffer solution (aCSF) within the bare and coaxial electrode regions.

In preparation for experiments, the bMEA and cMEA regions were characterized by measuring DC resistance (in air) between the individual electrodes for the bare electrode region and between all terminals (inner and outer electrode as well as inter-electrode) for the coaxial region. Typical resistances were in the GΩ range, indicating no shorts in the circuit. Capacitance of the coaxial samples was also measured and those results checked against the calculated values according to the equation for a coaxial capacitor:$c=2\pi l\varepsilon \;\text{ln}\;\left(r_{\text{oute}r}/r_{\text{inne}r}\right)$. Measured values were within 10% of the calculated values, with differences attributed, in part, to stray capacitance originating from the unshielded portions of the coaxes. Additionally, the devices needed to be sterilized prior to cell culture. This was done by placing them in a sterilization packet: a bag which contains a scaffold that expands to let steam pass to its inner contents during the sterilizing process and then contracts during a cooling phase to insulate the inside from any foreign contaminates. The packet was placed inside a steam autoclave and a standard dry process was run at 100°C for 30 min with a 30 min cool down phase). After the devices were autoclaved, they were placed inside a sterile hood until the HEK cells were ready to be plated (placed on the devices).

As mentioned earlier, optically-evoked field potentials were detected using HEK-293 transfected with the blue-light sensitive channelrhodopsin protein ChR2(H134R) (Lorentz, 1896). Transfection and culture processes, described in previous work (Naughton et al., 2016), were used. To ensure cell adherence to the bare electrode and coaxial structures contained in two separate PDMS wells, the two devices were incubated in a sterile solution of 0.01% poly-l-lysine overnight at 37°C 5% CO_2_. HEK-ChR2 cells were trypsinized from cell culture dishes and recovered by centrifugation at 595 g for 6 min at 4°C. The cells were resuspended in DMEM 10% FBS media containing 250 μg/ml G418 at a density of ~ 10^6^ cells/ml. A 0.1 ml aliquot of cells was added to one well of a coaxial device and cultured overnight at 37°C 5% CO_2_. The seeding density of cells almost completely covered both the bare electrode and coaxial structures within 24–48 h of subsequent cell culture and adherence. The color of the medium was carefully monitored to ensure cell health. From previous experiments, we noticed that dark yellow meant the medium needed to be changed and that there was cell overgrowth. Since we selected for cells of successful transfection, we wanted the entire pillar region to be covered in HEK293-ChR2 cells to ensure every sensing region was covered and therefore was a potential stimulation zone. Once it was evident there was cell overgrowth (yellow colored medium), the medium was aspirated and replaced with fresh medium. Immediately after this, the devices were covered in aluminum foil (to avoid exposure to stimulating light) and the devices were brought to the Multichannel Systems amplifier for measurement. A 473 nm DPSS laser (Model BL473-100FC ADR-700A, Shanghai Laser and Optics Century Co., Ltd.) coupled to a multimode 200 μm diameter optical fiber (0.39 NA, Thor Labs) with a spot size of ~350 μm was used for photo stimulation. Prior to placing the devices in the amplifier system, the laser light was characterized using the same process described in previous work (Naughton et al., 2016). The maximum intensity was found to be 20 mW/cm^2^ and this level was used throughout the experiment.

The bMEA was uncovered, placed in the amplifier system, and the macropads were aligned with the pins. A Ag/Cl pellet was placed into the electrolyte buffer solution to act as a ground, since no other ground was present in the area. All 60 channels were monitored simultaneously to ensure the baseline voltage reached a steady state for each sensing region. Initially, the data acquisition program was run continuously and the laser was aligned for topside illumination. The laser was manually actuated and the illumination area was moved throughout the entire sensing region. This was done to ensure a positive response from the cells. Once cell response due to optical stimulation was visually confirmed, the laser was adjusted and attached to a micromanipulator for backside illumination. The data acquisition program was changed to a trigger capture program using a TTL signal (Stimulus Generator STG4002, Multichannel Systems) with a 0.5 s square wave pulse. The laser was then moved to several sites below the area containing the individual sensing regions and a five sweep trial was performed at each spot (with averages displayed in Figure 4 and peak averages ± standard deviations in Figure 5). All 60 channels were monitored throughout each trial and the approximate laser location was noted prior to stimulation. Throughout the experiment, deflections could be seen in all illuminated working channels. For the bMEA region, a Ag/Cl pellet was again placed into the electrolyte buffer solution to act as a ground, since no other ground was present in the area. A pulse generator program was used to send in a train of 100 μV, 500 ms square-wave pulses spaced 1 s apart.

## Data Availability

The original contributions presented in the study are included in the article. Further inquiries can be directed to the corresponding author.
